# Gut-Derived Metabolite Phenylacetylglutamine and White Matter Hyperintensities in Patients With Acute Ischemic Stroke

**DOI:** 10.3389/fnagi.2021.675158

**Published:** 2021-07-30

**Authors:** Fang Yu, Xianjing Feng, Xi Li, Yunfang Luo, Minping Wei, Tingting Zhao, Jian Xia

**Affiliations:** ^1^Department of Neurology, Xiangya Hospital, Central South University, Changsha, China; ^2^Clinical Research Center for Cerebrovascular Disease of Hunan Province, Central South University, Changsha, China; ^3^National Clinical Research Center for Geriatric Disorders, Xiangya Hospital, Central South University, Changsha, China

**Keywords:** metabolomics, phenylacetylglutamine, white matter hyperintensities, ischemic stroke, biomarkers

## Abstract

**Background:** White matter hyperintensity (WMH) burden is associated with a higher risk of ischemic stroke. Phenylacetylglutamine (PAGln) is a gut microbiota-derived metabolite that may induce cardiovascular events by activating platelets and increasing the risk of thrombosis. The relationship between plasma PAGln and WMH burden in patients with ischemic stroke is unknown. This study was designed to investigate the association between plasma PAGln and WMH burden in patients with acute ischemic stroke.

**Methods:** A total of 595 patients with acute ischemic stroke were enrolled in this study within 14 days of symptom onset. The burden of WMH was evaluated using the Fazekas scale based on the fluid-attenuated inversion recovery sequence. The severity of overall WMH was defined as none–mild WMH (total Fazekas score 0–2) or moderate–severe WMH (total Fazekas score 3–6). Based on the severity of periventricular WMH (P-WMH) and deep WMH (D-WMH), patients were categorized into either a none–mild (Fazekas score 0–1) group or a moderate–severe (Fazekas score 2–3) group. Plasma PAGln levels were quantified using liquid chromatography–mass spectrometry.

**Results:** We found that patients with moderate–severe overall WMH showed higher plasma PAGln levels than patients with none–mild overall WMH, and similar results were found in the analyses according to P-WMH and D-WMH. The logistic regression analysis showed that the fourth PAGln quartile was independently associated with moderate–severe overall WMH (adjusted 95% CI 1.134–4.018) and P-WMH (adjusted 95% CI 1.174–4.226).

**Conclusion:** These findings suggest that higher plasma PAGln levels are associated with moderate–severe overall WMH and P-WMH in patients with acute ischemic stroke.

## Introduction

Stroke is a major cause of disability and death in China (Wu et al., 2019). White matter hyperintensity (WMH) is the most common radiological marker of small vessel disease (SVD) (Joutel and Chabriat, 2017), and mounting evidence has shown that WMH burden is related to the risk of first stroke, recurrent stroke, and poorer outcomes after stroke (Arsava et al., 2009; Park et al., 2019). Age and hypertension are widely considered to be the main risk factors for WMH (Rist et al., 2019), but they do not account for all the pathophysiological mechanisms of WMH. Therefore, identifying novel risk factors is crucial to improve our understanding of the etiology and consequences of WMH in patients with ischemic stroke.

Recently, altered circulating metabolites have been identified as contributing factors in stroke and cerebral small vessel disease (CSVD) (Nie et al., 2018; Janes et al., 2019). For instance, asymmetric dimethylarginine (ADMA) levels were found to be positively correlated with WMH burden in young asymptomatic patients (Janes et al., 2019). Phenylacetylglutamine (PAGln), a gut microbiota-derived metabolite, has been associated with adverse cardiovascular events, such as coronary artery disease and stroke (Nemet et al., 2020). PAGln is formed by the conjugation of glutamine and phenylacetate, which is derived from bacterial phenylalanine metabolism (Moldave and Meister, 1957). Higher plasma PAGln levels increase the risk of cardiovascular events which may be due to enhanced platelet activation and thrombosis potential (Nemet et al., 2020).

However, the relationship between circulating PAGln and WMH burden in ischemic stroke patients is unknown. Therefore, to enhance our knowledge of the predictive role of PAGln in WMH impairment, we prospectively investigated the relationship between circulating PAGln and WMH impairment in patients with ischemic stroke. This study represents the first cross-sectional study examining whether plasma PAGln levels are associated with WMH burden in ischemic stroke patients.

## Materials and Methods

### Study Participants

This study included consecutive patients with ischemic stroke confirmed between August 2017 and October 2020. We recruited 595 patients with ischemic stroke confirmed by diffusion-weighted imaging of the brain within 14 days of symptom onset. The other inclusion criterion was age ≥18 years. We excluded patients with disabilities (Modified Rankin Scale score ≥2) before stroke onset and those without fluid-attenuated inversion recovery sequence (FLAIR). This study was approved by the Ethics Committee of Xiangya Hospital. All participants provided written informed consent.

### Clinical Assessments

We assessed demographic characteristics and medical history, including age, sex, vascular risk factors [i.e., hypertension, diabetes mellitus, dyslipidemia, coronary heart disease (CAD), smoking, and drinking], based on the definitions previously described in detail (Feng et al., 2021). Complete blood count, liver and kidney function, blood glucose, homocysteine, and serum lipids were determined from overnight fasting venous blood samples from each participant on the second day of admission.

### Fluid-Attenuated Inversion Recovery Sequence Magnetic Resonance Imaging Assessment of WMH

Periventricular WMH (P-WMH) and deep WMH (D-WMH) were assessed on FLAIR images using the Fazekas scale, which ranges from 0 to 3. We categorized the severity of P-WMH and D-WMH as none–mild (Fazekas score 0–1) or moderate–severe (Fazekas score 2–3) (Yu et al., 2018). The total Fazekas score was classified based on the sum of P-WMH and D-WMH (range 0–6). The severity of overall WMH was identified as follows: none–mild WMH (Fazekas score 0–2) or moderate–severe WMH (Fazekas score 3–6) (Zhu et al., 2020).

### Quantification of PAGln

Overnight fasting venous blood samples were collected as soon as possible on the second day of admission. The whole blood sample was centrifuged into plasma and stored at −80°C until analysis. Plasma PAGln was quantified on an AB SCIEX TripleTOF 6500 system (AB SCIEX, Foster City, CA, USA) using liquid chromatography-mass spectrometry with D_5_-PAGln (CDN Isotopes, Cat # D-6900) as an internal standard. First, plasma was diluted 10-fold with ddH_2_O, then 2 μl of 1 ppm D5-PAGln was added to 48 μl of diluted plasma, and the mixture was diluted 4-fold with ice-cold methanol and vortexed for 1 min. The supernatant was then centrifuged at 21,000 × *g* at 4°C for 15 min and transferred to a clean vial for testing. Finally, 1 μl of the supernatant was injected into an Acquity UPLC BEH C18 column (Waters, Herts, UK) for analysis (50 × 2.1mm, 1.7 μm). The column temperature was 40°C, and the flow rate was 0.3 ml/min, with the mobile phase A containing 0.1% acetic acid in water and mobile phase B containing 0.1% acetic acid in water. We used known PAGln concentrations to establish a standard curve for the determination of PAGln concentrations. The PAGln concentration of the standard was 10 ng/ml. The intra-day coefficients of variation were 0.80–1.39%, and the inter-day coefficients of variation were 4.80–6.00%.

### Statistical Analysis

We used SPSS 22.0 (IBM Corp., Armonk, NY, USA) and GraphPad Prism 8 (GraphPad Software, San Diego, CA, USA) for the statistical analysis. The participants were dichotomized according to WMH burden into none–mild and moderate–severe groups using the Fazekas scores. In addition, participants were divided into four groups according to the quartiles of plasma PAGln concentrations. Categorical variables were described as proportions, and continuous variables were described as mean ± SD or medians [interquartile range (IQR)]. Continuous variables were compared using an ANOVA, Kruskal–Wallis test, or Mann–Whitney *U* test, as appropriate. Categorical variables were analyzed using the Pearson's χ^2^ test. We conducted a logistic regression analysis using the following three models: an unadjusted model, a model adjusted for age and sex, and a model adjusted for age, sex, and the variables showing *P* < 0.05 in the univariate analyses. We used the median to classify these confounding continuous variables in the regression analysis. Odds ratio (OR) and the 95% CI were obtained. A Spearman rank correlation was used to identify the association between plasma PAGln levels and Fazekas scores. The value of PAGln for the prediction of WMH severity was evaluated using a receiver operating characteristics (ROC) curve, and the area under the ROC curve (AUC) was calculated. All tests were two-sided. Statistical significance was set at *P* < 0.05.

## Results

### Clinical Characteristics of Patients With Ischemic Stroke

A total of 595 patients (67.7% male; median age, 61 years) with ischemic stroke were enrolled in our study. The median plasma PAGln level at admission was 2.06 μmol/L. Quartiles of PAGln levels were as follows: first quartile, <1.21 μmol/L; second quartile, 1.21–2.06 μmol/L; third quartile, >2.06–3.34 μmol/L; fourth quartile, >3.34 μmol/L. Higher PAGln quartiles were associated with high Fazekas scores, old age, high frequency of hypertension, diabetes mellitus, CAD, high levels of blood urea nitrogen and homocysteine, and low levels of estimated glomerular filtration rate (eGFR) (Table 1).

**Table 1:** Baseline characteristics of patients with ischemic stroke according to PAGln quartiles.

**Variables**	**First quartile**	**Second quartile**	**Third quartile**	**Fourth quartile**	***P* value**
	***n* = 149**	***n* = 149**	***n* = 149**	***n* = 148**	
Fazekas score	2.0 (1.5–4.0)	3.0 (2.0–4.0)	2.0 (2.0–4.0)	4.0 (2.0–5.0)	<0.001
Age (years)	54 (48–62)	60 (51–66)	63 (54–70)	67 (60–72)	<0.001
Sex (male, *N*, %)	95 (63.8%)	106 (71.1%)	103 (69.1%)	99 (66.9%)	0.562
HBP (*N*, %)	93 (62.4%)	113 (75.8%)	99 (66.4%)	119 (80.4%)	0.002
DM (*N*, %)	26 (17.4%)	47 (31.5%)	45 (30.2%)	54 (36.5%)	0.003
Hyperlipidemia (*N*, %)	46 (30.9%)	42 (28.2%)	40 (26.8%)	45 (30.4%)	0.855
CAD (*N*, %)	19 (12.8%)	15 (10.1%)	29 (19.5%)	39 (26.4%)	<0.001
Smoking (*N*, %)	68 (45.6%)	81 (54.4%)	71 (47.7%)	64 (43.2%)	0.253
Drinking (*N*, %)	48 (32.2%)	58 (38.9%)	59 (39.6%)	48 (32.4%)	0.372
SBP (mmHg)	140.0 (125.0–154.0)	144.0 (129.0–157.0)	143.0 (130.0–156.0)	142.5 (134.8–158.5)	0.135
DBP (mmHg)	84.0 (74.0–93.0)	82.0 (74.0–92.0)	82.0 (74.0–90.0)	81.0 (72.0–91.2)	0.632
BMI	23.5 (22.0–25.1)	23.6 (21.9–25.9)	22.9 (21.5–25.7)	23.0 (21.0–25.7)	0.678
White blood cell count (× 10^9^/L)	6.2 (5.2–7.9)	6.7 (5.7–8.2)	6.5 (5.5–8.1)	7.0 (5.8–8.1)	0.064
Platelet (× 10^9^/L)	208.0 (164.0–251.0)	209.0 (167.0–240.0)	196.0 (162.0–246.0)	203.5 (167.8–235.0)	0.615
BUN (mmol/L)	4.6 (3.9–5.6)	4.9 (4.1–6.0)	5.2 (4.1–6.2)	5.6 (4.6–7.2)	<0.001
eGFR (ml/min/1.73 m^2^)	89.4 (76.7–102.6)	86.6 (74.6–96.3)	81.5 (69.7–92.5)	72.7 (57.8–89.1)	<0.001
Uric acid (μmol/L)	335.9 (96.4)	341.1 (270.6–385.4)	307.3 (245.0–381.8)	332.3 (273.2–387.4)	0.175
TC (mmol/L)	3.9 (3.3–5.0)	4.4 (3.6–5.2)	4.2 (3.5–5.0)	4.3 (3.5–5.2)	0.110
TG (mmol/L)	1.5 (1.0–2.2)	1.6 (1.1–2.3)	1.5 (1.1–2.0)	1.5 (1.2–2.3)	0.525
HDL (mmol/L)	1.0 (0.8–1.2)	1.0 (0.8–1.2)	1.0 (0.9–1.2)	1.0 (0.9–1.1)	0.757
LDL (mmol/L)	2.4 (1.9–3.1)	2.6 (2.1–3.2)	2.6 (2.1–3.3)	2.7 (2.2–3.3)	0.078
Fasting blood-glucose (mmol/L)	5.4 (4.8–6.3)	5.8 (5.0–7.4)	5.9 (5.1–7.7)	5.7 (5.1–8.1)	0.065
HbA1c (%)	5.7(5.4–6.3)	5.9 (5.5–6.9)	5.9 (5.5–7.4)	6.0 (5.5–7.3)	0.017
Homocysteine (μmol/L)	12.3 (10.6–14.6)	13.4 (11.5–16.7)	13.2 (11.3–15.6)	14.7 (11.9–19.3)	<0.001

*PAGln, phenylacetylglutamine; HBP, hypertension; DM, diabetes mellitus; CAD, coronary artery disease; SBP, systolic blood pressure, DBP, diastolic blood pressure; BMI, body mass index; BUN, blood urea nitrogen; eGFR, estimated glomerular filtration rate; TC, total cholesterol; TG, triglycerides; HDL, high-density lipoprotein; LDL, low-density lipoprotein; HbA1c, glycosylated hemoglobin A1c*.

### The Association Between Plasma PAGln and the Severity of Overall WMH According to Total Fazekas Scores

**Figure 1:**
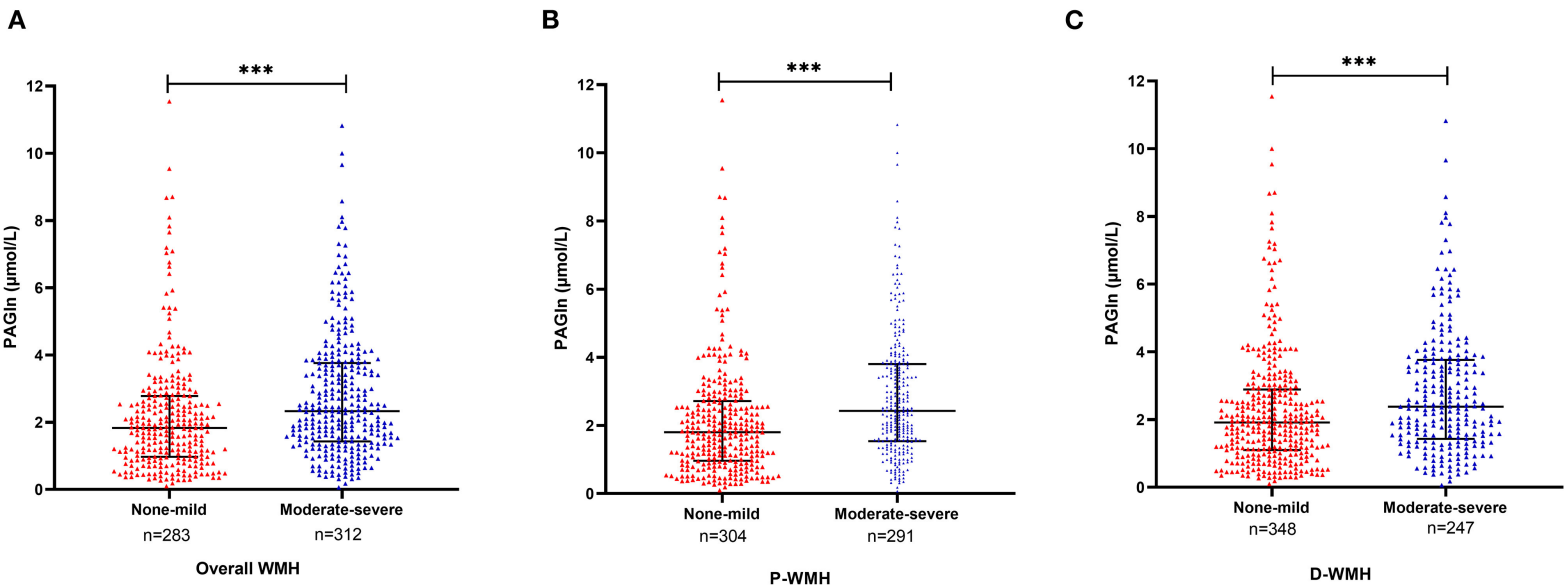
Plasma PAGln levels in different groups according to the severity of WMH. **(A)** Patients with moderate–severe overall WMH had higher plasma PAGln levels than patients with none–mild overall WMH [median 2.3 (IQR 1.5–3.8) vs. median 1.8 (IQR 1.0–2.8) μmol/L, *P* < 0.001]. **(B)** Patients with moderate–severe P-WMH had higher plasma PAGln levels than none–mild P-WMH group [median 2.4 (IQR 1.5–3.8) vs. median 1.8 (IQR 1.0–2.7) μmol/L, *P* < 0.001]. **(C)** Patients with moderate–severe D-WMH had higher PAGln levels than none–mild D-WMH group [median 2.4 (IQR 1.5–3.8) vs. median 1.9 (IQR 1.1–2.9) μmol/L, *P* < 0.001]. Horizontal lines represent median and interquartile ranges. WMH, white matter hyperintensity; PAGln, phenylacetylglutamine; P-WMH, periventricular white matter hyperintensity; D-WMH, deep white matter hyperintensity. Mann–Whitney *U* test, ^***^P < 0.001.

**Figure 2:**
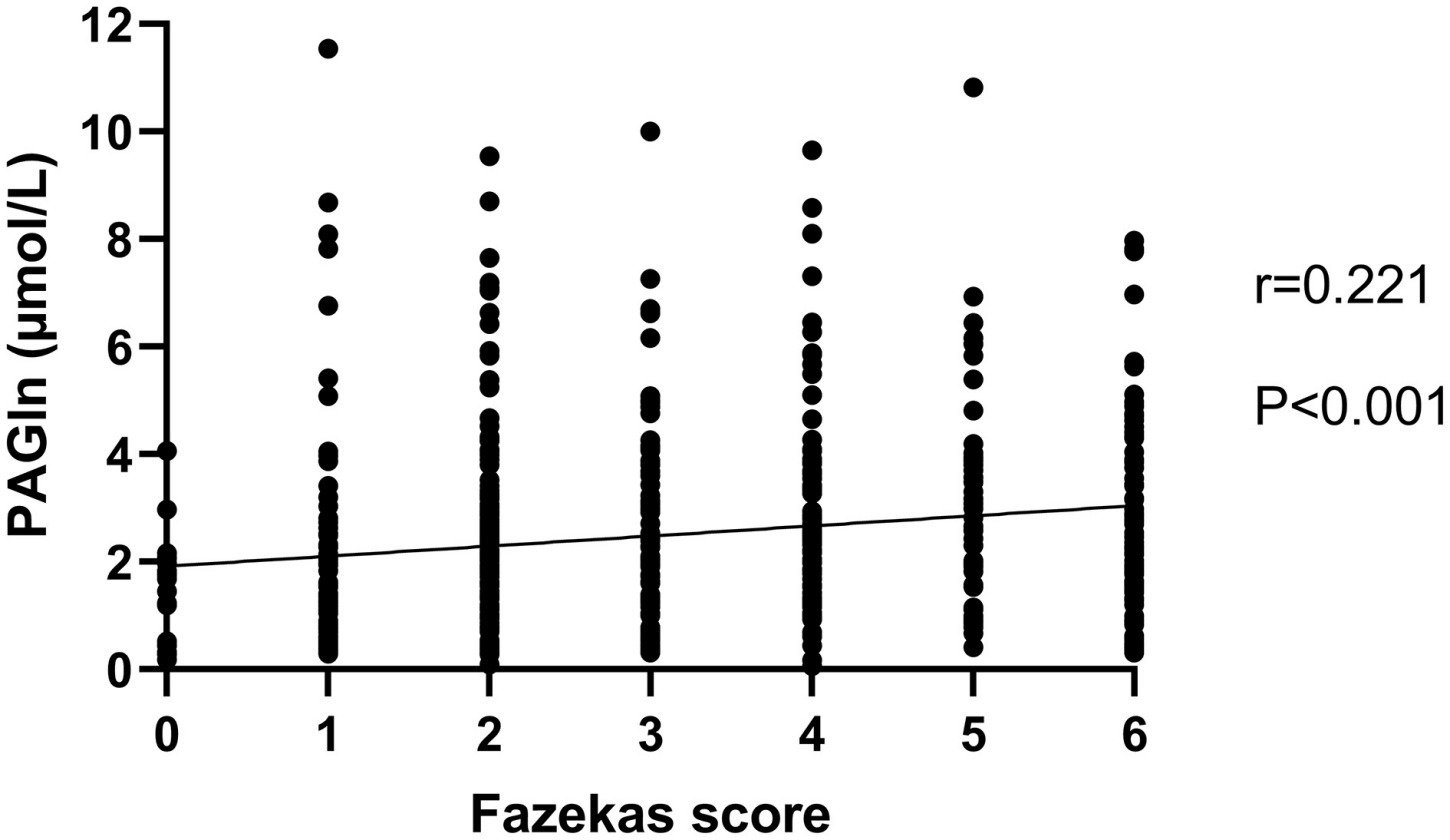
Correlation between PAGln levels and Fazekas score. PAGln levels showed a significant, although limited, relationship with the total Fazekas score (*r* = 0.221, *P* < 0.001, and Spearman rank correlation analysis). PAGln, phenylacetylglutamine.

There were 283 patients with none–mild overall WMH (total Fazekas score 0–2) and 312 patients with moderate–severe overall WMH (total Fazekas score 3–6). When compared with patients with none–mild WMH, patients with moderate–severe WMH were older and had a higher frequency of hypertension, diabetes mellitus, CAD, and higher levels of blood urea nitrogen and homocysteine. Lower levels of platelet count, eGFR, and total cholesterol were observed in moderate–severe WMH subjects (Table 2). We found higher plasma PAGln levels in patients with moderate–severe WMH than in patients with none–mild WMH [median 2.3 (IQR 1.5–3.8) vs. median 1.8 (IQR 1.0–2.8) μmol/L, *P* < 0.001] (Figure 1A). Moreover, PAGln levels showed a limited correlation with the Fazekas score (*r* = 0.221, *P* < 0.001) (Figure 2).

**Table 2:** Baseline characteristics of all patients according to the degree of overall WMH.

**Variables**	**None-mild WMH** ***n* = 283**	**Moderate-severe WMH** ***n* = 312**	***P* value**
Age (years)	55 (49–63)	66 (59–72)	<0.001
Sex (male, *N*, %)	200 (70.7%)	203 (65.1%)	0.144
HBP (*N*, %)	176 (62.2%)	248 (79.5%)	<0.001
DM (*N*, %)	70 (24.7%)	102 (32.7%)	0.032
Hyperlipidemia (*N*, %)	83 (29.3%)	90 (28.8%)	0.897
CAD (*N*, %)	33 (11.7%)	69 (22.1%)	<0.001
Smoking (*N*, %)	143 (50.5%)	141 (45.2%)	0.193
Drinking (*N*, %)	101 (35.7%)	112 (35.9%)	0.958
SBP (mmHg)	142.0 (127.5–154.0)	143.0 (130.0–159.2)	0.086
DBP (mmHg)	83.0 (74.0–93.0)	82.0 (73.0–91.0)	0.391
BMI	23.4 (22.0–25.2)	23.3 (20.9–25.8)	0.652
PAGln (μmol/L)	1.8 (1.0–2.8)	2.3 (1.5–3.8)	<0.001
White blood cell count ( ×10^9^/L)	6.7 (5.4–8.2)	6.6 (5.6–8.0)	0.946
Platelet ( ×10^9^/L)	208.0 (171.0–250.5)	199.0 (162.8–236.2)	0.043
BUN (mmol/L)	4.9 (3.9–6.0)	5.2 (4.2–6.3)	0.043
eGFR (ml/min/1.73 m^2^)	88.5 (74.5–98.6)	78.6 (64.5–90.2)	<0.001
Uric acid (μmol/L)	315.8 (272.9–380.1)	331.4 (268.3–391.9)	0.598
TC (mmol/L)	4.2 (3.4–5.2)	4.2 (3.5–4.9)	0.234
TG (mmol/L)	1.6 (1.2–2.2)	1.5 (1.0–2.2)	0.035
HDL (mmol/L)	1.0 (0.8–1.2)	1.0 (0.9–1.2)	0.318
LDL (mmol/L)	2.6 (2.1–3.4)	2.6 (2.0–3.1)	0.231
Fasting blood–glucose (mmol/L)	5.6 (5.0–7.1)	5.6 (5.0–7.7)	0.702
HbA1c (%)	5.8 (5.4–6.7)	5.9 (5.5–7.0)	0.100
Homocysteine (μmol/L)	12.7 (10.8–15.2)	13.8 (11.4–17.7)	0.002

*WMH, white matter hyperintensity; HBP, hypertension; DM, diabetes mellitus; CAD, coronary artery disease; SBP, systolic blood pressure, DBP, diastolic blood pressure; BMI, body mass index; PAGln, phenylacetylglutamine; BUN, blood urea nitrogen; eGFR, estimated glomerular filtration rate; TC, total cholesterol; TG, triglycerides; HDL, high-density lipoprotein; LDL, low-density lipoprotein; HbA1c, glycosylated hemoglobin A1c*.

The results of the logistic regression analyses are shown in Table 3. In the unadjusted model, when using the first quartile as a reference, the second and fourth quartiles of PAGln levels were independently associated with moderate–severe WMH (OR 2.212 and 95% CI 1.390–3.522 for the second quartile and OR 4.296 and 95% CI 2.639–6.994 for the fourth quartile). These results remained significant when adjusted for age and sex. When the multivariable model was further adjusted for age, sex, hypertension, diabetes mellitus, CAD, platelet counts, eGFR, triglycerides, and homocysteine levels, only the fourth quartile of PAGln level was independently associated with moderate–severe WMH (OR 2.134 and 95% CI 1.134–4.018).

**Table 3:** Logistic regression analyses of the association between PAGln levels and overall WMH.

	***P* value**	**OR**	**95% CI for OR**	
			**Lower**	**Upper**
**Unadjusted model**				
PAGln levels			
First quartile	Reference			
Second quartile	0.001	2.212	1.390	3.522
Third quartile	0.061	1.559	0.980	2.479
Fourth quartile	<0.001	4.296	2.639	6.994
**Adjusted model** ^**a**^				
PAGln levels			
First quartile	Reference			
Second quartile	0.020	1.818	1.098	3.010
Third quartile	0.755	0.921	0.549	1.544
Fourth quartile	0.009	2.053	1.195	3.528
Age (years)	<0.001	1.080	1.059	1.100
Sex (male)	0.781	0.946	0.642	1.396
**Adjusted model** ^**b**^				
PAGln levels			
First quartile	Reference			
Second quartile	0.085	1.670	0.932	2.994
Third quartile	0.963	0.986	0.547	1.778
Fourth quartile	0.019	2.134	1.134	4.018
Age (years)	<0.001	1.065	1.041	1.089
Sex (male vs. female)	0.324	0.776	0.469	1.285
HBP	0.399	1.235	0.757	2.015
DM	0.804	1.060	0.668	1.685
CAD	0.799	1.077	0.610	1.900
Platelet >204 ×10^9^ /L	0.195	0.755	0.494	1.155
eGFR ≤ 83.85 mL/min/1.73 m^2^	0.125	1.421	0.908	2.224
TG >1.52 mmol/L	0.262	0.785	0.514	1.198
Homocysteine >13.28 μmol/L	0.181	1.359	0.867	2.129

a *Adjusted model^a^: adjusted for age and sex.*

b *Adjusted model^b^: adjusted for age, sex, HBP, DM, CAD, platelet counts, eGFR, TG, and homocysteine levels*.

*WMH, white matter hyperintensity; OR, odds ratio; CI, confidence interval; PAGln, phenylacetylglutamine; HBP, hypertension; DM, diabetes mellitus; CAD, coronary artery disease; eGFR, estimated glomerular filtration rate; TG, triglycerides*.

### The Association Between Plasma PAGln and the Severity of WMH According to the Location of WMH

To further explore the relationship between plasma PAGln and different areas of WMH burden, we divided all patients into a P-WMH group and a D-WMH group. We categorized the severity of P-WMH and D-WMH as none–mild (Fazekas score 0–1) and moderate–severe (Fazekas score 2–3), respectively. There were 304 patients with none–mild P-WMH and 291 patients with moderate–severe P-WMH. Compared with patients with none-mild P-WMH, patients with moderate–severe P-WMH were older and had a higher frequency of hypertension, diabetes mellitus, and CAD, higher levels of blood urea nitrogen and homocysteine, and lower levels of eGFR and triglycerides. When classified by D-WMH, 348 and 247 patients were in the none–mild and moderate–severe D-WMH groups, respectively. Patients with moderate–severe D-WMH were more likely to have hypertension and CAD, higher systolic blood pressure, higher levels of blood urea nitrogen, high-density lipoprotein, and homocysteine, and lower levels of eGFR (Table 4).

**Table 4:** Characteristics of patients according to the scales of P-WMH and D-WMH.

**Variables**	**P-WMH**			**D-WMH**		
	**None–mild**	**Moderate–severe**	***P* value**	**None–mild**	**Moderate–severe**	***P* value**
	***n* = 304**	***n* = 291**		***n* = 348**	***n* = 247**	
Age (years)	55.0 (49.0–63.0)	67.0 (60.0–72.0)	<0.001	57.0 (50.0–64.0)	67.0 (59.0–72.0)	<0.001
Sex (male, *N*, %)	87 (28.6%)	105 (36.1%)	0.052	244 (70.1%)	159 (64.4%)	0.140
HBP (*N*, %)	191 (62.8%)	233 (80.1%)	<0.001	223 (64.1%)	201 (81.4%)	<0.001
DM (*N*, %)	73 (24.0%)	99 (34.0%)	0.007	98 (28.2%)	74 (30.0%)	0.633
Hyperlipidemia (*N*, %)	88 (28.9%)	85 (29.2%)	0.944	95 (27.3%)	78 (31.6%)	0.257
CAD (*N*, %)	37 (12.2%)	65 (22.3%)	0.001	44 (12.6%)	58 (23.5%)	<0.001
Smoking (*N*, %)	153 (50.3%)	131 (45.0%)	0.195	169 (48.6%)	115 (46.6%)	0.630
Drinking (*N*, %)	108 (35.5%)	105 (36.1%)	0.888	124 (35.6%)	89 (36.0%)	0.920
SBP (mmHg)	142.0 (127.0–155.0)	143.0 (130.0–158.5)	0.096	141.0 (129.0–154.0)	145.0 (130.0–161.0)	0.017
DBP (mmHg)	83.0 (74.0–94.0)	82.0 (73.0–90.0)	0.190	83.0 (74.0–92.0)	82.0 (73.0–91.5)	0.613
BMI	23.6 (22.0–25.2)	23.0 (20.8–25.8)	0.279	23.3 (21.9–25.6)	23.4 (20.9–25.7)	0.720
PAGln (μmmol/L)	1.8 (1.0–2.7)	2.4 (1.5–3.8)	<0.001	1.9 (1.1–2.9)	2.4 (1.5–3.8)	<0.001
White blood cell count ( ×10^9^ /L)	6.7 (5.4–8.2)	6.7 (5.6–8.0)	0.884	6.7 (5.5–8.1)	6.7 (5.5–8.0)	0.904
Platelet ( ×10^9^/L)	207.0 (167.8–249.0)	199.0 (163.0–239.5)	0.113	207.0 (167.8–249.0)	199.0 (163.0–239.5)	0.113
BUN (mmol/L)	4.9 (3.9–5.9)	5.2 (4.2–6.4)	0.009	5.0 (3.9–6.0)	5.1 (4.2–6.3)	0.033
eGFR (ml/min/1.73 m^2^)	88.5 (74.0–98.7)	77.7 (64.2–89.1)	<0.001	88.4 (74.6–98.2)	76.7 (62.2–88.2)	<0.001
Uric acid (μmol/L)	318.6 (273.1–385.7)	328.1 (267.1–385.8)	0.799	315.0 (272.3–379.1)	339.8 (271.2–393.2)	0.204
TC (mmol/L)	4.2 (3.4–5.2)	4.2 (3.4–4.9)	0.143	4.2 (3.4–5.2)	4.3 (3.5–5.0)	0.415
TG (mmol/L)	1.6 (1.2–2.2)	1.5 (1.0–2.2)	0.045	1.6 (1.1–2.2)	1.5 (1.0–2.3)	0.154
HDL (mmol/L)	1.0 (0.8–1.2)	1.0 (0.9–1.2)	0.669	1.0 (0.8–1.1)	1.0 (0.9–1.2)	0.004
LDL (mmol/L)	2.6 (2.1–3.4)	2.6 (2.0–3.1)	0.123	2.6 (2.0–3.3)	2.6 (2.1–3.2)	0.565
Fasting blood–glucose (mmol/L)	5.6 (4.9–6.9)	5.7 (5.0–7.7)	0.213	5.6 (5.0–7.4)	5.7 (5.0–7.3)	0.882
HbA1c (%)	5.8 (5.4–6.5)	6.0 (5.5–7.1)	0.314	5.8 (5.4–7.2)	5.9 (5.6–6.8)	0.647
Homocysteine (μmol/L)	12.7 (10.8–15.1)	14.0 (11.4–17.8)	<0.001	12.8 (10.8–15.5)	14.0 (11.6–18.3)	0.001

*P-WMH, periventricular white matter hyperintensity; D-WMH, deep white matter hyperintensity; HBP, hypertension; DM, diabetes mellitus; CAD, coronary artery disease; SBP, systolic blood pressure; DBP, diastolic blood pressure; BMI, body mass index; PAGln, phenylacetylglutamine; BUN, blood urea nitrogen; eGFR, estimated glomerular filtration rate; TC, total cholesterol; TG, triglycerides; HDL, high-density lipoprotein; LDL, low-density lipoprotein; HbA1c, glycosylated hemoglobin A1c*.

Levels of PAGln in the P-WMH and D-WMH groups are shown in Figures 1B,C. PAGln levels were elevated in patients with moderate–severe WMH. Binary logistic regression analyses demonstrated that the fourth PAGln quartile was independently associated with severe P-WMH (OR 2.227 and 95% CI 1.174–4.226) (using the first quartile as the reference) when adjusted for age, sex, hypertension, diabetes mellitus, CAD, blood urea nitrogen, eGFR, triglycerides, and homocysteine levels. However, the significant association between the second and fourth quartiles of PAGln levels with severe D-WMH disappeared when adjustments were made for age, sex, vascular risk factors, and laboratory biomarkers (Table 5).

**Table 5:** Logistic regression analyses of the association between PAGln levels and P-WMH and D-WMH.

	**P-WMH**					**D-WMH**			
	***P* value**	**OR**	**95% CI for OR**			***P* value**	**OR**	**95% CI for OR**	
			**Lower**	**Upper**				**Lower**	**Upper**
**Unadjusted model**					**Unadjusted model**				
PAGln levels					PAGln levels		
First quartile	Reference				First quartile	Reference			
Second quartile	0.003	2.021	1.263	3.235	Second quartile	0.022	1.748	1.083	2.823
Third quartile	0.024	1.719	1.073	2.755	Third quartile	0.177	1.396	0.860	2.266
Fourth quartile	<0.001	4.816	2.9491	7.867	Fourth quartile	<0.001	3.220	1.993	5.201
**Adjusted model** ^**a**^					**Adjusted model** ^**a**^				
PAGln levels					PAGln levels		
First quartile	Reference				First quartile	Reference			
Second quartile	0.062	1.637	0.976	2.747	Second quartile	0.171	1.428	0.858	2.379
Third quartile	0.983	0.994	0.585	1.689	Third quartile	0.629	0.878	0.518	1.488
Fourth quartile	0.004	2.247	1.297	3.890	Fourth quartile	0.065	1.648	0.970	2.799
Age (years)	<0.001	1.088	1.067	1.110	Age (years)	<0.001	1.068	1.049	1.088
Sex (male)	0.494	0.872	0.588	1.291	Sex (male)	0.684	0.924	0.633	1.349
**Adjusted model** ^**b**^					**Adjusted model** ^**b**^				
PAGln levels					PAGln levels		
First quartile	Reference				First quartile	Reference			
Second quartile	0.286	1.383	0.762	2.511	Second quartile	0.475	1.243	0.685	2.255
Third quartile	0.859	1.056	0.577	1.933	Third quartile	0.730	0.899	0.489	1.651
Fourth quartile	0.014	2.227	1.174	4.226	Fourth quartile	0.057	1.819	0.981	3.372
Age (years)	<0.001	1.077	1.051	1.103	Age (years)	<0.001	1.054	1.031	1.078
Sex (male vs. female)	0.377	0.796	0.480	1.321	Sex (male vs. female)	0.750	1.082	0.665	1.761
HBP	0.377	1.255	0.759	2.075	HBP	0.076	1.609	0.951	2.723
DM	0.414	1.215	0.761	1.939	CAD	0.724	1.103	0.640	1.901
CAD	0.974	0.990	0.561	1.749	SBP >142 mmHg	0.692	1.092	0.708	1.684
BUN >5.01 mmol/L	0.899	0.972	0.623	1.515	BUN >5.01 mmol/L	0.307	0.795	0.512	1.235
eGFR ≤ 83.85mL/min/1.73 m^2^	0.305	1.277	0.800	2.038	eGFR ≤ 83.85mL/min/1.73 m^2^	0.051	1.584	0.998	2.513
TG >1.52 mmol/L	0.154	0.733	0.479	1.123	HDL >1.00 mmol/L	0.006	1.847	1.196	2.853
Homocysteine >13.28 μmol/L	0.119	1.437	0.910	2.268	Homocysteine >13.28 μmol/L	0.260	1.303	0.822	2.067

a *Adjusted model^a^: adjusted for age and sex.*

b *P-WMH adjusted model^b^: adjusted for age, sex, HBP, DM, CAD, BUN, eGFR, TG, and homocysteine levels*.

*D-WMH adjusted model^b^: adjusted for age, sex, HBP, CAD, SBP, BUN, eGFR, HDL, and homocysteine levels.*

*WMH, white matter hyperintensity; P-WMH, periventricular white matter hyperintensity; D-WMH, deep white matter hyperintensity; OR, odds ratio; CI, confidence interval; PAGln, phenylacetylglutamine; HBP, hypertension; DM, diabetes mellitus; CAD, coronary artery disease; BUN, blood urea nitrogen; eGFR, estimated glomerular filtration rate; TG, triglycerides; HDL, high-density lipoprotein; SBP, systolic blood pressure*.

### Receiver Operating Characteristic Analyses of PAGln Levels According to the Severity of WMH

**Figure 3:**
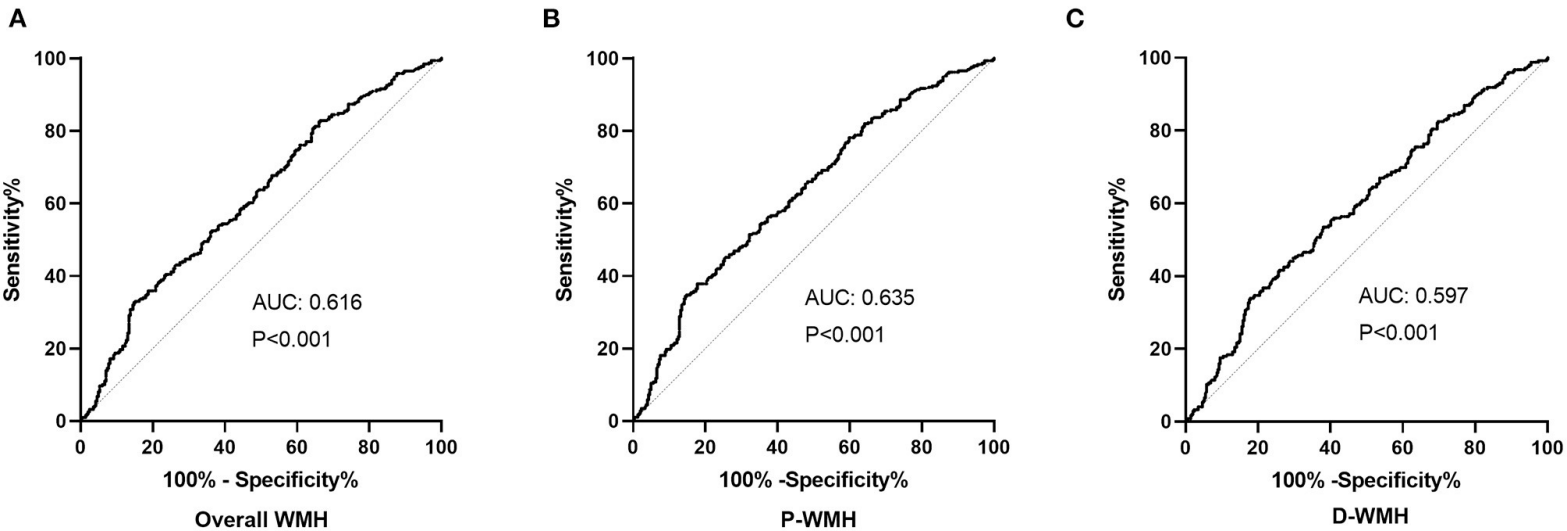
Receiver operating characteristic analysis of PAGln according to the severity of WMH. **(A)** The AUC was 0.616, and the optimal PAGln level cut-off value was 3.348 μmol/L for overall WMH. **(B)** The AUC was 0.635, and the optimal PAGln level cut-off value was 3.075μmol/L for P-WMH. **(C)** The AUC was 0.597, and the optimal PAGln level cut-off value was 3.341 μmol/L for D-WMH. ROC, receiver operating characteristic curve; PAGln, phenylacetylglutamine; WMH, white matter hyperintensity; P-WMH, periventricular white matter hyperintensity; D-WMH, deep white matter hyperintensity; AUC, area under the curve.

The diagnostic value of PAGln in distinguishing ischemic stroke patients according to WMH burden was evaluated using the ROC analysis. The AUCs for overall WMH, P-WMH, and D-WMH were 0.616, 0.635, and 0.579 (Figure 3), respectively. The optimal PAGln cut-off values were 3.348, 3.075, and 3.341 μmol/L for overall WMH, P-WMH, and D-WMH, respectively.

## Discussion

In this study, we conducted a targeted metabolomic analysis to explore the association between PAGln levels and WMH in patients with ischemic stroke. Our results demonstrated that plasma PAGln levels at admission were associated with the severity of WMH in patients with ischemic stroke. After adjusting for age, sex, and confounding factors, higher PAGln levels were independently associated with moderate–severe overall WMH. These associations were also found with P-WMH but not with D-WMH.

The pathophysiology of WMH remains unclear. Traditional vascular risk factors such as age, hypertension, diabetes mellitus, and smoking may play crucial roles in the pathological process of WMH and SVD (Rost et al., 2010; Giese et al., 2020). Previous studies have uncovered biomarkers of endothelial dysfunction, inflammation, and impaired fibrinolysis for WMH in stroke patients and the general population (Poggesi et al., 2016). Metabolomic biomarkers such as uric acid, homocysteine, AMDA, and ceramides have been reported to be related to WMH (Han et al., 2016). Of note, these altered metabolites might be involved in the pathological process of WMH through their common role in endothelial dysfunction. Many studies have investigated the role of microbiota in neurological disorders, but studies on WMH and SVD are relatively rare. Recently, Cai et al. reported on the role of the gut-immune-brain axis in arteriosclerotic SVD pathophysiology (Cai et al., 2021). Another cross-sectional study indicated that some microbiota may increase the risk of WMH and SVD (Saji et al., 2021).

Gut microbiota can produce metabolites or toxins that influence the health of the host. Gut microbiota-derived metabolites, such as trimethylamine-*N*-oxide (TMAO), tryptophan, and indole derivatives, may play critical roles in the pathogenesis of cardiovascular and cerebrovascular diseases (Ascher and Reinhardt, 2018; Wang and Zhao, 2018). TMAO has been the most studied gut microbiota-derived metabolite in recent years. Accumulating evidence has proven the causal links among TMAO, CAD, and stroke (Witkowski et al., 2020). Elevated TMAO and choline levels have recently been found to be associated with severe WMHs, especially P-WMH (Chen et al., 2021).

Phenylacetylglutamine, another gut microbial metabolite, has been reported to correlate with chronic kidney disease, diabetes mellitus, cardiovascular disease, and Parkinson's disease (Poesen et al., 2016; Urpi-Sarda et al., 2019; Shao et al., 2021). In 2020, Hazen et al. identified a causal contribution of PAGln to incident cardiovascular disease risks in a large sample clinical study (Nemet et al., 2020). This study suggested a clinical association between elevated PAGln levels and the overall burden of WMH and P-WMH. The possible mechanisms are as follows: first, studies have shown that PAGln levels are positively associated with age (Swann et al., 2013; Poesen et al., 2016), and our data also showed an increase in PAGln levels with increasing age, which is a known factor contributing to the pathology of WMH (Urpi-Sarda et al., 2019). Second, traditional vascular risk factors, such as hypertension and diabetes mellitus, are involved in the process of WMH (Tamura and Araki, 2015). As shown in Table 1, the group of patients with higher PAGln levels had higher rates of hypertension and diabetes, and studies have also suggested higher PAGln levels in patients with diabetes (Nemet et al., 2020), and therefore the relationship between PAGln and WMH might be due to the underlying mechanism of small vessel abnormalities of hypertension and diabetes (Tamura and Araki, 2015). Our data showed a decrease in eGFR with increasing PAGln levels, and previous observations also showed that kidney impairment measured by eGFR was strongly associated with high serum PAGln levels (Wang and Zhao, 2018). Furthermore, we found that decreased eGFR was associated with moderate–severe WMH, consistent with the previous results (Steinicke et al., 2012; Zong et al., 2016).

In this study, WMH was divided into P-WMH and D-WMH. A limited number of studies have investigated the differences between P-WMH and D-WMH; however, the underlying mechanism has not yet been fully elucidated. Our results suggest the involvement of PAGln in the development of P-WMH, but not D-WMH, and the detailed mechanisms require further investigation. Previous pathology studies have shown that P-WMH is more likely to be associated with inflammation and chronic hypoperfusion, whereas D-WMH is related to ischemic damage (Fazekas et al., 1993). These differences may provide possible explanations for the relationship between PAGln and P-WMH. Previous studies have found a relationship between PAGln levels and human immunodeficiency virus-associated dementia and impaired cognitive function in patients receiving hemodialysis (Cassol et al., 2014; Kurella Tamura et al., 2016). As a uremic metabolite, PAGln can lead to blood–brain barrier disruption and impair P-WMH. In addition, there are some controversies regarding the relationship between diabetes and P-WMH and D-WMH. In our data, we found a higher rate of diabetes in patients with moderate–severe P-WMH; however, no difference was found in patients with D-WMH. Limited studies (Urpi-Sarda et al., 2019; Nemet et al., 2020) have revealed the associations between diabetes and PAGln levels. The closer relationship between diabetes and P-WMH might be the reason why PAGln is associated more with P-WMH than D-WMH.

There were some limitations to this study. First, this was a cross-sectional study, so we could not establish a causal relationship between PAGln and WMH. Second, participants in our study were recruited from a single center, and this could have led to patient selection bias. Third, PAGln levels were only analyzed at a single time point, and information on dynamic changes in PAGln was missing. Fourth, investigations of the gut microbiota were lacking in this study. Finally, we used a less precise visual rating scale to assess the degree of WMH. Quantification of WMH is needed to further investigate the relationship between PAGln and WMH volume.

## Conclusion

In conclusion, higher plasma PAGln levels might be a biomarker of moderate–severe WMH, especially moderate–severe P-WMH. Further studies concerning the cause–effect relationship between PAGln and WMH are needed.

## Data Availability Statement

The datasets generated for this study are available on request to the corresponding author.

## Ethics Statement

The studies involving human participants were reviewed and approved by Xiangya Hospital Ethics Committee. The patients/participants provided their written informed consent to participate in this study.

## Author Contributions

FY and XF: methodology and writing—original draft preparation. XL, YL, MW, and TZ: investigation and data curation. JX: conceptualization and writing—reviewing and editing. All authors contributed to the article and approved the submitted version.

## Conflict of Interest

The authors declare that the research was conducted in the absence of any commercial or financial relationships that could be construed as a potential conflict of interest.

## Publisher's Note

All claims expressed in this article are solely those of the authors and do not necessarily represent those of their affiliated organizations, or those of the publisher, the editors and the reviewers. Any product that may be evaluated in this article, or claim that may be made by its manufacturer, is not guaranteed or endorsed by the publisher.
